# Recent advances in cerebral cavernous malformation research

**DOI:** 10.20517/2574-1209.2018.34

**Published:** 2018-08-28

**Authors:** Akhil Padarti, Jun Zhang

**Affiliations:** Department of Biomedical Sciences, Texas Tech University Health Science Center El Paso, El Paso, TX 79905, USA

**Keywords:** Cerebral cavernous malformation, cerebral cavernous malformation signaling complex, angiogenesis, endothelial cells, cellular function, microvessel lesions, protein structure, function domain, motif

## Abstract

Cerebral cavernous malformations (CCM) are manifested by microvascular lesions characterized by leaky endothelial cells with minimal intervening parenchyma predominantly in the central nervous system predisposed to hemorrhagic stroke, resulting in focal neurological defects. Till date, three proteins are implicated in this condition: CCM1 (KRIT1), CCM2 (MGC4607), and CCM3 (PDCD10). These multi-domain proteins form a protein complex via CCM2 that function as a docking site for the CCM signaling complex, which modulates many signaling pathways. Defects in the formation of this signaling complex have been shown to affect a wide range of cellular processes including cell-cell contact stability, vascular angiogenesis, oxidative damage protection and multiple biogenic events. In this review we provide an update on recent advances in structure and function of these CCM proteins, especially focusing on the signaling cascades involved in CCM pathogenesis and the resultant CCM cellular phenotypes in the past decade.

## INTRODUCTION

Cerebral cavernous malformations (CCMs) are vessel dilatations within microvascular beds in the brain that are predisposed to hemorrhagic stroke. These microvascular malformations are present in 0.5% in the general population^[1]^. These lesions are characterized by densely packed tortuous microvessels outlined with deficient interstitial brain parenchyma^[2,3]^, increasing the propensity of these vascular lesions for leakage^[4]^. These microvascular lesions are predominantly found in the central nervous system (CNS) but are also known to affect skin and liver. Although it is highly prevalent^[1]^, a vast majority (approximately 70%) are asymptomatic^[5]^.

Even in patients affected by disease within the same family, there is a wide range of clinical presentations which are primarily determined by the number and size of lesions. This suggests that additional genetic modifiers or non-genetic factors may exist in the pathogenesis of the disease^[6]^. As of date, *CCM1* (*KRIT1*) on chromosome 7q, *CCM2* (*MGC4607/OSM/Malcavernin*) on chromosome 7p, and *CCM3* (*PDCD10/TFAR15*) on chromosome 3q are genes that are known to cause the familial form of CCM. It is estimated that 1 in 200 people harbor a potential mutation in one of three *CCM* genes and the frequency increases to 1 in 70 in the Hispanic population^[7]^. An epidemiological study showed that 94% of all familial forms and 57% of all sporadic forms of CCM result from mutations in one of these three genes^[8]^, raising a possibility that mutations in other genetic loci might exist^[9,10]^. Recently, many polymorphisms were found in patients with the sporadic form of the disease, further strengthening the argument that the presence of other genetic risk factors that can contribute to the sporadic cases of CCM is possible^[11]^. However, endeavors to identify novel *CCM* loci have so far failed. It was proposed that the lesions formed in the familial form through a “second hit” mutation while sporadic vascular lesions occurred due to somatic mutations in both alleles caused by mutagens, radiation, or other factors. However, the familial form has an increased propensity for lesions since only one functional allele is present. Therefore, the familial form characteristically develops neurological manifestations earlier with multiple brain lesions, while the sporadic form develops late neurological manifestation with solitary brain lesions^[12,13]^.

As mentioned earlier, at least one of these three genes (*CCM1*, *2*, *3*) is disrupted in most CCM cases in humans^[14]^. The three CCM proteins interact to form a protein complex^[15–19]^ which further interacts with other proteins^[19–22]^. This CCM protein complex, referred to as the CCM signaling complex (CSC)^[19]^, has affinity toward a wide range of ligands and such interactions are involved in cell adhesion, migration, and apoptosis^[15,16,19,23–25]^. Current evidence suggests that as core CSC proteins, CCM proteins act as scaffolds for many signal molecules and spatiotemporally regulate localization and activity of these proteins, with none possessing innate catalytic activity^[26]^. Homozygous mutations in any of the three CCM proteins are nonviable, indicating their essential role in biogenesis as phenotype suppressors^[11–13]^. We will mainly focus on these three core CSC proteins in this review.

## CLINICAL PRESENTATION

All forms of human *CCM* mutations induce lesions in the CNS. These lesions are used to follow the course of disease (clinically symptomatic or not) both in the laboratory and in the clinical setting through MRI. A CCM variant observed in the skin tissue is known as hyperkeratotic cutaneous capillary venous malformation (HCCVM)^[27]^. Other cutaneous manifestations include café-au-lait spots, cutaneous venous malformation, and cavernous hemangiomas^[28,29]^. Genetic analysis showed that HCCVMs were only found in patients with a frameshift mutation resulting in a premature stop codon in exon 1 of *CCM1*. *CCM1* mutations have also been associated with hepatic angiomas^[30]^. As for clinical manifestation, CCM1 and CCM2 familial forms are similar while CCM3 familial form has several unique characteristics. *CCM1* and *CCM2* mutations result in spinal cavernous angiomas^[28]^. Unlike *CCM1* and *CCM2* mutations which manifest later, *CCM3* mutations manifest as early as age 20^[31]^. CCM3 cases have the heaviest disease burden characterized by numerous CNS lesions, with an increased risk of bleeding^[32,33]^. In addition to cutaneous manifestations with premature termination codon mutation in exon 1 of *CCM1*^[34]^, *CCM3* mutation cases also present scoliosis^[33]^, mental retardation^[35]^, and meningiomas^[31]^. No other genetic locus for familial forms of CCM has been identified yet^[36]^. However, there are still patients suffering from CCM with normal *CCM1*, *CCM2*, and *CCM3* genetic screen results. Even utilizing next-generation sequencing to screen the whole genome of CCM patients with known and unknown mutations, no causative mutation among all three *CCM* loci was detected for these CCM patients^[37]^, further suggesting the existence of a potential new *CCM* locus.

## PROTEIN STRUCTURE

CCM1 is the largest of the three CCM proteins (736 amino acids) and the most common *CCM* gene mutated^[7,38]^. 50% of all familial forms of CCM are due to mutations of *CCM1*^[36,39]^. The penetrance of *CCM1* mutation is around 88%^[40]^. A recurrent *CCM1* missense mutation (Q455X) is prevalent in the Hispanic population, hence it is named common Hispanic mutation^[7]^. It is present in high frequency in southwestern US^[41]^. From N-terminal to C-terminal, CCM1 protein contains a NUDIX domain, three NPxY/F motifs, Ankyrin repeat domain, a FERM domain, nuclear export signal (residues 551–562)^[18,25,42]^ and nuclear localization signals (residues 46–51, 569–572)^[23,42–44]^.

### CCM1 C-terminal FERM domain

Located at the C-terminus (residues 420–736), the FERM domain contains three subdomains (F1–F3). The F1 subdomain of the FERM domain folds into an ubiquitin-like fold, while the F2 subdomain folds into an acyl-CoA binding fold. Several proteins are known binding partners to this region of CCM1^[45]^. The F3 subdomain of FERM domain, a bona fide PH domain, binds to NPXY motifs. There have been several reports on this type of interaction in CCM1^[19,46–48]^. Some reports indicate that the C-terminal FERM domain binds to its first NPXY motif intramolecularly, thereby allowing the CCM1 protein to adopt a closed conformation. They further suggested that in the open conformation, the CCM1 binds to ICAP1α and localizes it to the cytoplasm and to the nucleus. In the closed conformation, the protein’s binding site is inaccessible to ICAP1α and results in increased CCM1 binding to microtubules through the NUDIX domain, retaining ICAP1α in the cell membrane. In support of this theory, it was further suggested that cellular binding partners of CCM1 to the F1/F2 subdomain of the FERM domain or NUDIX domain may drive this conformational change in CCM1^[47,48]^. However, new evidence showed that unlike most N-terminal FERM domain-containing proteins, CCM1 (containing a C-terminal FERM domain) does not seem to follow the auto-inhibition mechanism to undergo an intramolecular folding. In fact, both dynamic light scattering (DLS) and native protein gel electrophoresis showed an increased tendency of CCM1 to undergo CCM3 - like oligomerization through intermolecular binding between F3 subdomain of FERM domain and 3 NPXY motifs among partnering CCM1 proteins over intramolecular binding of F3 subdomain of FERM domain - NPXY motifs within CCM1^[19]^.

### CCM1 ankyrin repeat domain

The ankyrin repeat domain (ARD) domain (residues 288 and 419) in CCM1 is composed of 4 ankyrin repeats. This ARD packs onto the N-terminal side of the F1 subdomain of the FERM domain. Each repeat is identical and composed of two α-helices joined by a β-hairpin. The binding site is between the α-helices. These four Ankyrin repeats stack vertically into an “L”-shaped fold. The fourth repeat contains a tripeptide insert (G401, N402, and N403) in the binding site stabilized by a conversed W404. This tryptophan residue sits in a highly conserved hydrophobic pocket. X-ray crystallography showed that the ARD domain is bound tightly to the FERM domain. This highly conserved interaction is particularly mediated by the convex surfaces of Ankyrin repeats 2 and 3 in ARD domain and the β2 strand and α2 helix in F1 subdomain in the FERM domain. This interaction is stabilized by 10–12 hydrogen bonds over 993 A2. The ARD domain was termed as the F0 subdomain in the FERM domain due to its proximity to the FERM domain. The presence of another domain in such as position has been found in other FERM domain - containing proteins, i.e., Talin and Kindlin^[49,50]^. Many proteins such as RAP1 and HEG1 are able to bind to the FERM domain with little to no structural change of the ARD domain required. The ARD in CCM1 is different from other ARD in proteins such as DARPins due to its inability to bind to β-tubulin. CCM1 was suggested to be a tubulin binding protein, however both structural studies and binding assays show that the ARD domain is not utilized for this interaction^[51,52]^. Till now, there are no known binding partners to the ARD domain in CCM1.

### Multiple NPXY motifs in CCM1 protein

CCM1 also contains 3 NPXY motifs (residues 192–195, NPAY; 231–234, NPLF; 250–253, NPYF) in the central portion of the protein which provide important interactions with phosphotyrosine binding (PTB), PH, FERM domains, *etc*. The first motif, which is the only one that can be phosphorylated, has a remarkably strong binding affinity to ICAP1α^[20,25]^. NPXY motifs 2 and 3 can only bind to DAB-like PTB domains, including CCM2^[18,19]^.

### CCM1 NUDIX domain

The NUDIX domain is found in the N-terminus (residues 1–170) of CCM1 and it contains a stretch of basic residues that potentially interacts with microtubules. NUDIX domains are usually found in hydrolases that bind to a variety of substrates. The NUDIX fold in CCM1 is a centrally positioned β-sheet with flanking α-helices. Traditionally, the NUDIX domain contains a NUDIX box which contains the Gx_5_Ex_7_REUxEExGU motif^[53]^. However, CCM1 doesn’t contain a traditional NUDIX motif, yet the tertiary structure of N-terminus of CCM1 still adopts a NUDIX fold. The catalytic residues found in other NUDIX domains are missing in the CCM1 NUDIX domain^[54]^. Therefore, the function of the NUDIX domain in CCM1 may be different from other NUDIX domain-containing proteins. Despite the similarities, superimposition of the X-ray crystal crystallography of the NUDIX domain with known substrates (81 in total) revealed no potential binding partners. Therefore, the function of this domain is still uncertain^[54]^. However, sequence analysis has elucidated the presence of several known sequences such as potential tubulin binding sequence^[48]^ and a nuclear localization sequence (NLS)^[42]^ within the NUDIX domain.

CCM2 is the second largest of the CCM proteins, 444 amino acids in length and contains a PTB domain at the N-terminus and a harmonin homology (HH) domain at the C-terminus^[3]^. 20% of all familial forms of CCM are due to mutations of CCM2^[36,39]^, however, the penetrance of CCM2 mutation was reported to be 100%^[40]^. CCM2 is found ubiquitously expressed in the endothelial cells (EC) from various organs^[55,56]^. Despite the lack of a recognizable NLS and NES, CCM2 is found in both the nucleus and cytoplasm due to its interaction with CCM1^[18,23,57]^. In the absence of functional CCM1, CCM2 is not localized to the cell junction, however, this function is recovered with the addition of wild-type CCM1, implying that binding to CCM1 is essential for localization of CCM2 to the cell junction^[58]^.

### CCM2 HH domain

CCM2 functions as the scaffold in the CSC with binding sites for both CCM1 and CCM3^[16]^. The HH domain at the C-terminus (residues 283–375) is comprised of 6 packed α-helices termed H1*, H1, H2, H3, H4, and H5 in that order from N-terminus to C-terminus. The H1* is a short α-helix with 3 amino acid residues and H4 is a 3_10_ helix that contains 13 residues. This domain is stabilized by several intramolecular interactions (i.e., R346 to E314, R354 to E366, and P355 to F356). This C-terminal domain bears structural similarity to harmonin protein and therefore termed HH domain. Although there is structural similarity, CCM2 HH domain is able to bind to neither Cadherin 23 (a validated binding partner of Harmonin) nor to CCM3. This HH domain exists in two conformations, monomeric and dimeric. The dimeric form has an increased affinity for dimerization; however, there are no sufficient data to affirm the occurrence of dimeric CCM2 *in vivo* yet^[45]^.

### CCM2 PTB domain

The N-terminus of the CCM2 contains a DAB-like PTB domain^[59]^. This domain contains 2 β-sheets composed of 7 β-strands, with α-helices capped at both ends. It was shown that this PTB domain binds to the NPXY motifs present in CCM1. Yeast two-hybrid assays showed that CCM2 PTB domain was able to bind to a CCM1 construct that contained the second and third NPXY motifs but not the first motif^[17,18]^.

CCM3 is the smallest of the 3 CCM proteins with 212 amino acids. CCM3 mutations tend to result in the most aggressive form of the disease^[8]^. 10% of all familial forms of CCM are due to mutations in *CCM3* gene^[36,39]^. The penetrance of *CCM3* mutation is approximately over 60%^[40]^. In one study that sought for promoter variants for the *CCM* genes, two protective single nucleotide polymorphisms were identified in the promoter region of CCM3 (rs9853967 and rs11714980) to be associated with CCMs, while no causative variants were identified in the promoter regions of CCM1 or CCM2, among the selected CCM patient cohort. These variants could partially explain the range of disease burden seen in CCM^[60]^. CCM3 is localized to the *cis*-face of the Golgi body^[61]^. Its interactions with phospholipids, PtdIns^[3–5]^, were believed to facilitate the translocation of CCM3 to the plasma membrane^[62]^. CCM3 is a 2-domain-containing protein where both domains are conjoined by a flexible hinge region^[63]^. The tertiary structure of CCM3 is a V-shaped structure. After some invertebrate analogs of CCM3 were identified and studied, it was found that CCM3 is the most evolutionarily conserved of the three CCM proteins^[64,65]^. However, CCM1 and CCM2 are predominantly found in the vertebrates^[63,66]^.

### CCM3 N-terminal dimerization domain

CCM3 exists as a homodimer in the cell due to presence of the dimerization domain. It is made up of four α-helices: α1, α2, α3, and small α4. These four α-helices interlock with another set of four α-helices from a partner CCM3 dimerization domain. This dimerization domain is also used to bind to GCKIII kinases forming a heterodimer. The interactions between GCKIII and flexible hinge regions of CCM3 control the equilibrium between CCM3 homodimer and CCM3-GCKIII heterodimer^[45]^.

### CCM3 C-terminal focal adhesion targeting - homology domain

The C-terminus of the CCM3 protein contains a focal adhesion targeting-homology domain (FAT-H) domain. This domain is commonly found in some tyrosine kinases such as Pyk2 and FAK. It is also composed of four α-helices: α5, α6, α7, and α8. This domain is utilized to bind to large variety proteins including CCM2 and phosphotidylinositides^[62]^.

## NEWLY IDENTIFIED CELLULAR COMPONENTS OF CSC COMPLEX

CCM1 is highly expressed in EC cells during embryogenesis^[67]^. It is localized ubiquitously throughout the cell including the nucleus^[17,20,38,42,68]^.

### CCM1 C-terminal FERM domain

CCM1 was shown to bind to a small GTPase Rap1 utilizing its FERM domain through X-ray crystallography^[43,44]^ and yeast two-hybrid^[69]^. This interaction localizes CCM1 to the periphery of the cell to facilitate signaling at the cell-cell junctions^[48]^. In contrary, the release of CCM1 from the plasma membrane is due to its interaction with ICAP1α. This complex localizes to the nucleus^[17]^. Rap1 is a GTPase that has many cellular functions such as maintaining cell-cell contacts and integrin-mediated cell adhesion^[70]^. Rap1 is activated by GTPase-activating proteins and CCM1 has a large affinity towards this activated form^[71]^. Disruption of RAP1 and CCM1 interaction results in the absence of CCM1 localization to the cell membrane particularly the adherens junction^[71]^. Superimposition of Rap1 and CCM1 showed that no conformational change was required for bindings to occur^[52]^. The FERM domain contains 3 subdomains F1, F2, and F3. RAP1 binding site in the FERM domain overlaps F1 and F2 subdomains. HRas is another GTPase that binds to the same binding pocket as RAP1. However, in competition assays, RAP1 binds more strongly to CCM1 over HRas due to specific residues in the F2 subdomain of CCM1. This stronger binding affinity to RAP1 is dependent on the interaction of K570 on CCM1 with E45 on RAP1, with respective mutations significantly decreasing binding. Further analysis of the X-ray crystallography of CCM1 and RAP1 showed that the switch II region in RAP1 doesn’t contribute to any binding affinity. This is a result of the interaction between Y419 on CCM1 and F64 on RAP1 which destabilizes the switch II region^[43]^. Rap1 acts as an inhibitor of CCM1 interaction with microtubules^[48]^. The inhibition mechanism is the binding of Rap1, releasing CCM1 from the cell membrane and spatially blocking the interaction between CCM1 and microtubules leading to an overall increase in stability of the cell junction^[72]^. Rap1 is found in mice in two forms: Rap1a and Rap1b. Individual deletions of either protein have minimal effect on embryogenesis. However, a combined deletion is embryo lethal. It results in the perineural vessel dilation and hemorrhage, demonstrating the importance of Rap1 in EC cells maturation and angiogenesis. *Rap1* deletion was shown to reduce VEGFR2 signaling^[73]^. Unlike *CCM1* deletions which result in malformed branchial arches, the formation of patent branchial arches can still be seen in *Rap1* deletion, suggesting that CCM1 acts along alternate pathways for vessel formation^[74]^. CCM1 FERM domain also binds to HEG1 protein, which further enables CCM1 localization at the cell junction. HEG1 is a transmembrane protein that contains an NPxY motif on its cytoplasmic tail. The binding pocket of HEG1 in CCM1 does not overlap with RAP1. The binding strength of HEG1 to CCM1 is independent of binding to RAP1. HEG1 interacts with the hydrophobic pocket between F1 and F3 subdomain. The interaction between HEG1 and CCM1 is trifold: F1 subdomain through polar interactions, F3 subdomain through hydrophobic interaction, and α2A helix between F1 and F3 subdomain. Analysis of electron density shows that the C-terminal dipeptide on HEG1 of tyrosine-phenylalanine is critical for their binding. This binding is not in the category of the classical PH (F3 subdomain) - NPxY motif interaction. In fact, the binding pocket is similar to the inositol phosphate binding site in the PH domain^[75]^. Similar to the binding of RAP1, the binding of HEG1 doesn’t illicit any conformational change in the FERM domain^[43]^. The binding of CCM1 with HEG1 and RAP1 is essential for appropriate cardiovascular development, and disruption of these interactions was shown to cause the phenotype of CCM^[71]^. *Heg1*-null zebrafishes manifest with the same cardiovascular phenotype as Ccm1 null mutants^[76]^. Infusion of CCM1 mutants lacking the binding ability to RAP1 and HEG1 into *Ccm1* null zebrafish could not reverse the cardiovascular phenotype^[71,76]^, indicating the essential role of the interaction.

### Multiple NPXY motifs in CCM1 protein

CCM1 binds to ICAP1α and acts a competitive inhibitor of ICAP1α and β1-integrin interactions[20,68]. ICAP1α contains a PTB domain that binds to β1-integrin and modulate β1-integrin mediated cellular function^[15,23–25,54]^. Mutagenesis of the T778 and V790 or N792 and Y795 of the cytoplasmic tails of β1-integrin prevents binding to ICAP1α. However, the exact consequence of this interaction is not fully agreed upon. The overwhelming theory is that CCM1 acts to sequester ICAP1α resulting in increased levels of β1-integrin activation because ICAP1α is a potent repressor of β1-integrins^[15,23–25,54]^. However, one study suggests that CCM1 interaction with ICAP1α stabilizes ICAP1α, therefore increasing β1-integrin activation. This effect is profound when low levels of CCM1 results in paradoxically increased β1-integrin activation.However, appropriate amount of β1-integrin is necessary for development of vascular sinusoids^[77]^, cell cycle^[78,79]^, and bone development^[80,81]^. ICAP1α PTB domain is a DAB-like PTB domain where the interaction between ICAP1α and CCM1 is independent of the phosphorylation status of the NPXY motif. Binding of CCM1 or β1-integrin to ICAP1α doesn’t result in any structural changes^[82]^. CCM1 binding to ICAP1α through a bidentate interaction with the ICAP1α PTB domain. This interaction is identical to ICAP1α and β1-integrin interaction thereby resulting in competitive inhibition^[15,23–25]^. CCM1 utilizes the first NPXY motif and a RR region to bind to ICAP1α PTB domain^[83]^. The RR region is a novel site that binds to the N-terminus of NPXY motif. This N-terminal region adopts an α-helix confirmation that binds to the loop between β1 and β2 and well as β5 and β6 and α1 helix of the PTB domain^[82]^. The interaction between the PTB domain and the RR site is mediated by the conserved arginine residues on the CCM1 (R179 and R185), binding to polar side chains such as the carbonyl groups of D146, D93, and Q96 on ICAP1α. The interaction of the PTB domain and the NPxY motif is mediated by the interaction of N192 and Y195 on CCM1 to the ICAP1α binding site of PTB domain, L135, I138, I139, and C184. Mutation in the NPXY binding sites on ICAP1α inhibits binding to cytoplasmic tail of β1 integrin. However, mutation in the RR binding sites (D146, D93, and Q96) did not affect binding with cytoplasmic tail of β1 integrin. Therefore, unlike CCM1-ICAP1α interaction, the binding between ICAP1α and β1-integrin is not a bidentate interaction^[54]^.

CCM1 binds to CCM2 and SNX17 through the utilization of the second and third NPXY motifs^[18,19,59,84]^. Biochemical studies demonstrate that CCM2 utilizes its PTB domain to bind to the third NPXY motif of CCM1. Several leucine residues in CCM2 (L113, L115, L155, L198, and L213) are paramount for adequate binding to CCM1. Several residues downstreaming to the NPXY motif such as V244 and V248 are also important. Even a conservative mutation like V244L was found to significantly decrease binding. An X-ray crystallography of CCM2 with the third NPxY motif of CCM1 was determined. However, Co-IP shows that both the second and third NPxY motifs are required to bind to CCM1^[18]^. Additionally, biochemical studies do not show an increased affinity with a construct containing both the second and third NPxY motifs over just the third NPxY motif^[59]^.

### CCM2 PTB domain

Some PTB domains can bind to phospholipids and it was expected that such an affinity in CCM2 PTB domain will transport CCM2 complex to the plasma membrane, however no consensus has been reached in that regard. One study stated that CCM2 PTB domain is able to bind to phospholipids^[85]^, while other studies suggested the opposite^[3,19]^. CCM2 PTB domain has been shown to interact with two proteins, TrkA and Smurf1. TrkA is present in neurons. The PTB domain of CCM2 binds to the cytoplasmic tail of TrkA^[86]^. This complex binds with CCM3 and STK25 to form a large complex. This may happen through a direct mechanism where CCM2 brings CCM3 or an indirect mechanism. However, the formation of this complex leads to apoptosis. This pathway is found to be especially important in the field of prognostic and therapeutic aspects of neuroblastoma and medulloblastoma^[87]^. CCM2 PTB domain was also reported to bind to Smurf1 HECT domain. This interaction brings Smurf1 to the plasma membrane^[88]^. This interaction has been related to RhoA degradation.The lack of RhoA degradation results in uncurbed activation of Rho-associated coiled coil-forming kinase (ROCK) which leads to stress fiber formation^[89]^. One report stated that CCM2 protein in macrophages is found adjacent to newly synthesized actin polymers. This localization of CCM2 is compatible with the predicted function of regulating RhoA because RhoA is active in actively synthesizing actin polymers^[22]^.

### CCM2 C-terminal HH domain

A four nucleotide duplication of the last exon of CCM2 was introduced to create a mutation disrupting the structure of the HH domain at the C-terminus. This mutated form of CCM2 protein was able to bind to CCM1 and CCM3, however unable to bind to MEKK3, a Map3 kinase, suggesting that the HH domain mediates the interaction between CCM2 and MEKK3^[90]^.

### CCM2 KARET domain and LD motif

Some reports showed that CCM2 interacted with CCM3 through a KARET domain at the C-terminus of CCM2 (residues 228–444), which overlaps with HH domain^[16,17,63,86,87]^. However, it was recently shown through pull-down assays that the binding pocket was located in the middle of CCM2 (residues 223–238), between the N-terminal PTB domain and C-terminal HH domain. This region was determined to contain a LD motif, which binds to FAT-H domain on CCM3. The FAT-H domain of CCM3 contains a hydrophobic pocket termed HP1 which is the binding site for CCM2. The LD motif adopts a 3.5 turn α-helix parallel to α7-helix in CCM3. The interaction is largely made up of hydrophobic residues from CCM2 (T225, I226, F228, L229, A232, I233, G236, and A237) binding to hydrophobic residues from CCM3 (I131, I134, A135, I138, L142, V168, F174, L178, S171, K132, and K139). These hydrophobic resides are also extremely conserved through evolution, indicating their significance^[63]^. CCM2-CCM3 complex formation reciprocally protects the proteins from degradation. Either CCM2 or CCM3 depletions result in concurrent decreases in the reciprocal protein. Furthermore, CCM2 and CCM3 mutated cells grew slower than wild-type ones. This mutant phenotype can be rescued with the addition of CCM2 or CCM3. Overexpression of CCM2 in CCM3-depleted cells did not restore cellular proliferation, however overexpression of CCM3 in CCM2-depleted cells did, indicating that the CCM3 may have a greater contribution to cell proliferation^[91]^.

### CCM2-Like protein

Recently CCM2-Like (Ccm2l) protein was identified in zebrafish. This protein bears large sequence similarity to CCM2. It contains a PH domain as opposed to a PTB domain, found in CCM2. Our lab showed that PTB domains and PH domains can have overlapping abilities^[19]^. Despite the similarities, the function of Ccm2l and CCM2 are not identical. The N-terminus of CCM1, which contains the NPxY motifs, can bind to Ccm2l, potentially through the PH domain. Although, it is still unknown which of the three NPxY motifs are used by Ccm2l for binding. Ccm2l, which is selectively expressed in EC^[92]^, sequesters CCM1 by acting as a competitive inhibitor, however, it is unable to bind to CCM3. The ability to bind to CCM1 results in the inhibition of CCM2 mediated junctional stability. During embryogenesis, Ccm1 is expressed in the notochord while Ccm2 is expressed heavily in tissue anterior to the notochord. Interestingly, Ccm2l is found in both regions. Despite these differences, Ccm2l knockout zebrafish exhibits cardiac phenotype, i.e., cardiomegaly. This phenotype is seen to a milder extent in *Ccm1*, *Ccm2*, and *Heg1* null mutants. The effect of Ccm2l is more pronounced in the heart than large vessels. In fact, there is partial phenotypic rescue with Ccm2 over-induction in Ccm2l-null zebrafish. Therefore, it is concluded that Ccm2l acts in the Heg-Ccm pathway^[93]^. It is still unclear whether CCM2L is involved in the pathogenesis of CCM^[26]^.

### CCM3 dimerization

The N-terminus of one CCM3 can bind to another CCM3 in the native state, driven by identical hydrophobic residues, L44, A47, I66, and L67^[94]^. An important set of binding partners of CCM3 are GCKIII kinases such as Mst4 and Stk25. These kinases contain an N-terminal catalytic domain and C-terminal regulatory domain. The C-terminal regulatory domain of GCKIII can bind to dimerization domain in CCM3. The tertiary structure of the C-terminus of GCKIII is similar to the N-terminus of CCM3 and can compete with and replace a CCM3 in the CCM3 homodimer. These kinases adopt an independent V-shaped domain as well. Each side of the V in the GCKIII protein is made up of several α-helices. Mutations in the hydrophobic residues that removed CCM3 dimerization effectively also inhibit GCKIII and CCM3 binding^[95]^. However, CCM3 has higher binding affinity to GCKIII kinases over CCM3 homodimer. There exists a long linker peptide in CCM3 between α3 and α4 helix along the CCM3 protein. In the homodimer state, this region folds into α-helix and merges with α4 forming an extended helix. This extended helix is stabilized by hydrophobic residues found in the antiparallel α1 helix in the partner CCM3 within the homodimer. Hydrophobic residues (V72, F76, L80, M83) in the extended α4 helix interact with hydrophobic residues (V25, A24, P21, M20, V18, and M17) in α1 helix of partner CCM3. This stabilizing interaction doesn’t occur in the GCKIII-CCM3 complex, because the corresponding α1 helix in GCKIII doesn’t contain any hydrophobic residues. In the interaction between CCM3-GCKIII, the linker region between α3 and α4 helices in CCM3 is less structured and able to adopt a flexible conformation. This allows hydrophobic residues (F76, L80, and M83) in α4 helix in CCM3 to fall into a hydrophobic pocket formed by α1 and α3 helix in CCM3 through intra-molecular binding. This twisting of the CCM3 causes in the full N-terminal face of CCM3 to interact with the full C-terminal face of GCKIII, which doesn’t occur in the homodimer of CCM3 due to the inherent bend in the CCM3 tertiary structure^[63,96]^. Therefore, there is a larger binding area between CCM3 and GCKIII. This larger-interface interaction present results in a much higher binding affinity^[95]^.

Knockout models of Stk24 and Stk25 caused cardiovascular disease similar to that observed in CCM3 knockout models^[97]^. STK25, a serine/threonine kinase, controls RhoA activation, which is a GTPase. Loss of RhoA results in stress fiber formation^[98]^. It has been well documented that CCM phenotype causes increased stress fiber formation; however several reports contradicted this finding^[99,100]^. Increase RhoA activation leads to increased ROCK. ROCK is another serine/threonine kinase that phosphorylates several proteins: myosin light chain, MLC phosphatase, and LIM kinase. MLC phosphatase decreases the cross linking of myosin and actin, the source of fiber contractility. Phosphorylation by ROCK inhibits MLC phosphatase. In contrast, ROCK dependent phosphorylation of LIM kinase results in activation. Active LIM kinase catalyzes phosphorylation of cofilin, which inhibits cofilin activity that regulates actin depolymerization. Both pathways, LIM kinase and MLC phosphatase, lead to stress fiber formation. ROCK inhibition results in regression of stress fiber^[26]^. Increases in ROCK activity have been recorded in CCM lesions. Interestingly, increases in ROCK activity have been seen in histologically normal blood vessels in CCM1 deficient mice, suggesting possible involvement of ROCK signaling in the pathogenesis of CCM lesions^[101]^.

### CCM3 C-terminal FAT-H domain

CCM3 contains a FAT-H domain at the C-terminus that is used to bind to CCM2. The surface of the domain contains a hydrophobic patch termed hydrophobic patch 1 (HP1), which is found between α7 and α8 helix. This is the site for binding various proteins such as CCM2^[63]^, striatins^[61]^, and paxillin^[102]^. For interaction of both stratins and paxillin, CCM3 recognizes LD motifs that adopt helical structures. Compared to CCM2-CCM3 complex, CCM3-paxillin complex has a smaller surface area, the LD motif is smaller (~ 2 turns), and the LD motif helix is less parallel to α7 helix in CCM3^[91]^. Paxillin is known to bind to FAT-H domains in FAK and Pyk2^[63]^. Through fluorescence images, it was determined that paxillin and CCM3 were co-localized to the plasma membrane at the leading edge^[102]^. The functionality of this binding is still unknown, one hypothesis is that the formation of some CCM3-GCKIII complexes is under the control of paxillin phosphorylation. Therefore, paxillin may be sequestering CCM3 from activity^[103]^.

## CELLULAR SIGNAL TRANSDUCTION

### CCM1 plays a role in Notch signaling

Cells with increased CCM1 activity show overexpression of HEY1 and DLL4, two major players in notch signaling. Notch signaling increases PI3K/AKT signal pathway and activated AKT leads to suppression of ERK1/2 by dephosphorylation. CCM1 deficient cells and CCM lesions show increased phosphorylation of ERK1/2^[104]^. AKT phosphorylation is also important in regulating the expression of SOD2, which is an important free radical scavenger in the cell. SOD2 is upregulated with increases in reactive oxygen species through AKT phosphorylation. In the absence of CCM1, AKT phosphorylation is decreased^[72,105]^, leading to decreased expression of SOD2, and therefore increasing oxidative damage in the cell^[106]^. CCM1 is also an inducer of SOD2 through interaction of ND1. ND1 is an important actin stabilization protein that binds to CCM1. This interaction increases the expression of SOD2. Therefore, CCM1 can prevent oxidative damage and cell death through complex induction of SOD2^[107]^.

### CCM1 involved in KLF4/KLF2 signaling pathways

Both *in vivo* studies with *Ccm1* knockout mice and *in vitro* studies with CCM1 silencing in human brain EC (hCMEC) showed elevation of KLF4 nuclear signal. KLF4 has been reported to play an important role in EC in biogenesis of veins and angiogenesis in general. Combined silencing of both KLF4 and CCM1 significantly decreases the disease mortality (75% reduction of mouse mortality) and modest improvement of vascular lesions (reduced vascular density in retina). The prototypical lesions in CCM lack mesenchymal intervening tissue, which is due to increased proliferation and dysfunctional migration, both of which are mediated with KLF4 inhibition. In this signaling pathway, CCM1 binds and sequesters MEKK3, which in turn activates MEK5, which subsequently activates ERK5^[108,109]^. ERK5 is a known inducer of KLF4 in EC cells^[110–112]^. KLF4 is a transcription factor which activates BMP6 and decreases SMAD1 phosphorylation. It has been shown that SMAD is activated in CCM deficient condition and leads to active BMP6 and TGF-β. These two downstream proteins mitigate the histological manifestation of CCM, i.e., lack of intervening parenchyma^[113]^. KLF2 is another transcription factor that is induced by a similar signaling cascade^[114]^. KLF2 is responsible for the cardiac manifestations and increased angiogenesis seen in CCM^[115,116]^. KLF4/KLF2 are transcription factors that suppress expression of thrombospondin1 (TSP1) which functions as an angiogenic inhibitor^[117]^. Loss of TSP1 was found to exacerbate CCM phenotype in *Ccm1* deficient mice. Abnormalities of cell-cell junction are found to be the initial manifestation of CCM1. It was found that decreases in Claudin-5 and ZO-1 levels were the first to be observed before changes in VE-cadherin levels. The perturbed expression of these cell junction proteins can be rescued in *Ccm1* knockout mice with exogenous TSP1 (3TSR). 3TSR was found to decrease both VEGR2 phosphorylation leading to decreased angiogenesis and increased TGF-β activation. Furthermore, this treatment decreases the lesion burden in mice. Therefore, there is a possibility to utilize KLF4 inhibitors, ERK5 inhibitors, or exogenous TSP1 for potential therapeutic applications in CCM in the near future^[118]^.

### CCM1 is a key player in integrin signaling

β1-integrin signaling is an important regulator in many cellular functions such as cellular migration and adhesion^[15,24,25,57]^. These functions are especially important for EC cells. ICAP1α is a repressor of β1-integrin signaling^[46]^. Our previous results indicate that CCM1 binds to ICAP1α and modulates ICAP1α and β1-integrin interaction. Depletion of CCM1 and ICAP1α synergistically inhibits extracellular signal-regulated kinase/mitogen-activated protein (ERK/MAP) kinase pathway activation on cell survival^[15,23–25,57,72]^. We hypothesized that CCM1 regulated recruitment of ICAP1α to the cell membrane in proximity to focal adhesions, which may be critical for the maintenance of cellular architecture as well as regulation of β1-integrin-mediated signaling^[25]^. The follow-up studies found that ICAP1α deficiencies result in many osteoblastic defects which are the direct results of β1-integrin activation. CCM1 acts as a competitive inhibitor for the interaction of ICAP1α and β1 integrin. The lack of inhibition of ICAP1α leads to excessive inhibition of β1-integrin which is thought to cause the leaky vasculature found in CCM^[54]^. CCM1 has been reported to be responsible for localization of ICAP1α to the nucleus, through the use of the N-terminus NLS^[42]^. It was shown that only in the presence on intact CCM1-ICAP1α interaction and functional NLS in CCM1 does ICAP1α localize to the nucleus^[47]^. However, ICAP1α also contains a NLS and drives CCM1 localization to the nucleus. In the absence of ICAP1α, CCM1 is evenly spread throughout the cell, but in the presence ICAP1α, the CCM1 localizes to the nucleus. Alanine walking in NLS1, NLS2, and NES in CCM1 showed that only NLS1 affected CCM1 localization. NLS2 and NES mutation showed identical CCM1 localization to wild-type CCM1. However, functional ICAP1α is able to translocate NLS1 mutated CCM1 into the nucleus. However, the N-terminus of CCM1 is unable to translocate ICAP1α with a deficient NLS. The addition of CCM1 to ICAP1α-silenced cells results in CCM1 accumulation only in the cytoplasm, suggesting that ICAP1α drives localization of CCM1, not the other way around^[119]^.

### CCM proteins modulate VEGF signaling

Mutations in CCM1 and CCM3 were found to increase translocation of β-catenin from the cytosol to the nucleus. This leads to increased expression of various proteins such as VEGF-A, which can be further reversed by the addition of a β catenin transcription inhibitor. The increased level of VEGR-A activates VEGFR2, which is shown by increased VEGFR2 phosphorylation. This increases the endothelial cell (EC) permeability, leading to vascular leakage. This phenotype can be rescued with the addition of VEGFR2 inhibitors in both *in vitro* and *in vivo* conditions. Interestingly, VEGF inhibition blocked the formation of stress fiber formation. Therefore, the stress fiber formation is caused to some extent by VEGF signaling. Furthermore, enhanced VEGF signaling results in increased cellular migration. A wound-healing assay showed that CCM1 deficient cells had a 25% increase in migration compared to the cultured cells treated with VEGF. However, this phenotype can be reversed in the *CCM1*-null cells with the treatment of VEGF inhibitors. VEGFR2 phosphorylation results in downstream phosphorylation of β-catenin and VE-cadherin, which results in disruption of interaction with α-catenin^[120]^ and p120 catenin^[121]^ respectively. This results in translocation of β-catenin into the nucleus for further downstream effects. However, VEGF inhibitors were not sufficient to inhibit the β-catenin and VE-cadherin disassociation seen in *CCM1* deficiency, suggesting that other mechanisms are involved for the disassociation^[122]^.

### CCM2 modulates MAPK signaling

CCM2 leads to downstream activation of p38 MAPK, which is upregulated in osmotic shock. It is still unclear for the role of the CCM2 in the p38 MAPK activation pathway. One report stated that CCM2 localizes to the cell membrane where it facilitates binding MEKK3 and RAC1 leading to activation of MAPK^[17,22]^. Another report showed that CCM2 is able to bind to F-actin, suggesting that CCM2 forms a complex that links RAC1-dependent actin reorganization to p38 MAPK signal pathway^[123]^. Another report showed that the signaling pathway is through phospholipase C (PLC). The complex of CCM2-RAC1 causes a change in PLC cascade, leading to MAPK activation^[124]^. While another report showed that CCM2 affects the JNK and MKK signaling leading to an alternative pathway to promote MAPK activation^[89]^.

### CCM3 plays a role in Notch signaling

In recent years, several mechanisms for the pathogenesis of CCMs have been proposed such as decreased Notch signaling^[125]^, increased VEGF signaling^[126]^, or increased ERK activity in the deficiency of CCM3^[127]^. It has been recently reported that CCM3 affects EC function by regulation of DLL4^[128]^. Down-regulation of CCM3 resulted in decreased expression of DLL4 and Notch4, but no change was observed in Notch1. This was shown in both cell lines and brain tissue in CCM patients. In fact, the vascular phenotype found in *CCM3* mutants can be replicated through mutations in DLL4. Aberrant DLL4/notch signaling results in overexpression of vasodilators during angiogenesis. The phenotype of hyperangiogenesis in CCM3 deficient condition can be rescued by overexpression of DLL4. Abnormal Notch signaling leads to many downstream effects. Notch signaling is also reported to be involved in the regulation of expression of VEGF receptors, to modulate vascular bed architecture^[129]^ and angiogenesis^[39,130]^. In fact, *CCM3* deficient cells increase the expression levels of VEGF which affects cell survival through ERK1/2 activity^[131]^. ERK1/2 kinase was reported to be upregulated in *CCM3* deficient lesions, which can be reversed when DLL4 function is rescued through induction of recombinant DLL4. Therefore, CCM3-mediated notch signaling also affects ERK1/2 and VEGF functions leading to abnormalities in EC cells. The finding that CCM3 deficient phenotype can be rescued through DLL4 overexpression creates a promising venue for future pharmacotherapy^[126]^.

### TLR4 signaling in CCM pathogenesis

It was found that induction of gram negative bacterial abscesses in Ccm1 and Ccm2 deficient mice significantly increased the phenotypic severity of CCM lesions. The effect was increased in mice with hematogenous infections of gram-negative bacteria. In fact, exposure to just lipopolysaccharide (LPS) was sufficient for significant lesion formation in the mice. It is well known that LPS response is mediated by TLR4 pathway^[132]^ and that MEKK3 deficiency terminates the signal^[133]^. MEKK3-KLF2/4 pathway has already been implicated in CCM lesion formation^[90]^, which is further validated by decreased expression of KLF2/KLF4 with an LPS injection, suggesting the existence of TLR4-MEKK3-KLF2/4 pathway. Furthermore, heterozygous TLR4 mutants had a significantly decreased CCM lesion formation and homozygous mutants had a complete resolution of CCM lesions. This suggests that TLR4 signaling drives CCM lesions development. In fact, genetic polymorphisms in TLR4 (rs10759930) and CD14 (rs778587) (a TLR4 co-receptor) that result in increased expression of respective proteins do result in increased CCM lesions. These findings were further supported by lack of CCM lesion formation in mice that were surgically removed as fetuses and grown in sterile conditions. Therefore, exogenous stimulation of TLR4 may be involved in CCM lesion formation. Two additional experiments were also performed to prove post-natal suppression of lesion can be achieved: (1) TLR4 antagonists can decrease lesion severity in mice through decreased TLR4 signaling; (2) the course of antibiotics also decreases lesion severity. These antibiotics altered the nature of the microbiome in the mice for decreased TLR4 stimulation. This report provides a new avenue for potential CCM pharmacotherapy^[134]^.

## CELLULAR FUNCTIONS AND BIOGENESIS

### Angiogenesis

CCM lesions are hallmarked by abnormally increased EC proliferation. There are many proposed mechanisms for angiogenesis implicated in CCM. ICAP1α is involved in NOTCH signaling. Therefore, loss of CCM1 or ICAP1α results in increased angiogenesis^[104]^. CCM3 is also reported to be involved in NOTCH signaling resulting in increased angiogenesis^[126]^. CCM proteins also modulate MAP kinase activity which in turn modulates angiogenesis^[89]^. It is also reported that CCM3 increases VEGF receptor concentrations, thereby resulting in increased EC proliferation^[135]^. However, there is still controversy of the mechanistic pathways for control of angiogenesis by the CCM proteins^[23,136]^.

### Microvascular integrity

CCM lesions are not only hallmarked by the presence of abnormal EC proliferation and migration but also increase the leakage predisposing the lesions to hemorrhage. There are several explanations for this phenomenon. ANKS1B is a novel PTB domain containing protein that binds to the third NPXY motif in CCM1. No change of EC cell proliferation, migration or sprouting was observed in *ANKS1B*-deficient EC, suggesting that *ANKS1B*-deficiency did not affect the CCM1 activity or Notch signaling. However, these EC cells had decreased transendothelial resistance (TER), which cannot be rescued by ROCK inhibitors. This observation indicates that ANKS1B regulated EC cell adhesion without involving RhoA signaling. Further, increased TER was achieved by overexpression of ANKS1B in CCM1 depletion, indicating that *CCM1* is not involved in ANKS1B signaling either^[137]^. Another explanation is that the increased vascular permeability is due to stress fiber formation in CCM lesions. CCM proteins are responsible for inhibiting Rho kinase. However, unopposed activation of Rho kinase results in increased phosphorylation of myosin light chain, which was also seen in CCM lesions^[58]^. Another theory is the dysfunction of RAP1 and CCM1. CCM1 acts as a scaffold for RAP1 and is important for cell junction protein β-catenin and VE-Cadherin, thereby stabilizing the cell membrane. VE-Cadherin is a part of the adherens junction^[138]^. Therefore, dysfunction of VE-Cadherin affects cell contacts. β-catenin is a nuclear factor. Dysfunction of CCM proteins results in increased translocation of β-catenin in the nucleus that lead to EC proliferation^[139]^.

The vascular permeability seen in *Ccm1* knockout mice can be rescued by SOD2 and catalase infusions with antibody-mediated targeting to the endothelium. TNF-α was able to induce vascular permeability in the arterioles in these rescued mice. However, TNF-α is unable to induce vascular permeability in *Ccm1* null mice. It was also shown that in the absence of CCM1, TNF-α was unable to generate ROS. This suggests that TNF-α function involves CCM1. Yet, CCM lesions have shown to have elevated ROS. This is a result of increased activity of NADPH oxidases (NOX). Both *in vitro* and *in vivo* CCM lesions showed significant up-regulation of NOX4. NOX4 is not only a source of ROS, but also an enhancer for downstream activation of NF-κB. This can be rescued by treatment of NOX4 inhibitors. Furthermore, broad spectrum NF-κB inhibitor (i.e., N-(E)-p-coumaroyl-3-hydroxyanthranilic acid, YAv1) inhibits NF-κB activation due to NOX4 or TNF-α, which can rescue the defective endothelial barrier seen in CCM^[140]^. These inhibitors could potentially be utilized for CCM pharmacotherapy in the future.

### CCM1 protects cells from oxidative damage

All three CCM proteins follow a knudsonian pattern of inheritance. Therefore, a second mutation is necessary for development of lesions and reactive oxidative species are a source of DNA mutations. Initially, Ccm mouse models were made with Ccm1 and Ccm2 heterozygous mutant mice. However, these mice never showed any CCM lesion phenotype, so another mutation, Msh2, was developed into these mice. Msh2 is a DNA damage-repair protein that decreases DNA mutations. Only mice with heterozygous Ccm1 and homozygous Msh2 deletion showed considerable vascular CCM lesions^[101]^. Ccm1 is a regulator of FoxO1, through an unknown mechanism. This transcription factor induces the transcription of Sod2 and Sirt1, two important anti-oxidants in the cell^[106]^. It was also shown that CCM1 can regulate Rho GTPase, by interacting with ND1-L in the presence of oxidative stress^[107]^. Another potential pathway modulated by CCM1 to limit oxidative stress is JNK/c-Jun signaling. In *CCM1*-null cells, there is an overexpression of C-Jun which can be reversed by reintroduction of CCM1. Therefore, CCM1 protects the cells from downstream oxidative stress of C-Jun redox pathways^[141]^. The cells lacking CCM1 also have increased activity of COX-2, a mediator of inflammatory pathways in the cells^[142]^. This is consistent with the *in vivo* data that *Ccm1* knockout mice had hyper-exaggerated response to inflammatory agents^[143]^. Therefore, it can be concluded that inflammation and oxidative stress are involved in CCM lesion formation^[144]^. CCM1 provides protection against oxidative stress in the cell by utilizing anti-inflammatory and anti-oxidant pathways^[106]^.

CCM is associated with increased ROS that mediate cellular damage. The cell usually responds through up-regulation of anti-oxidant enzymes. *CCM1* depletion induced lesions show increased levels of Nrf2 transcription factor and Glo1 enzyme, both having important anti-oxidant functions in the cell. This results in a paradoxical increase in cell death due to protective mechanisms. However, chronically activated anti-oxidant mechanisms result in impairment of regular redox reactions in the cell. Many other vascular diseases have overactivation of Nrf2^[145,146]^. It was shown that chronic Nrf2 activation as seen in CCM is paradoxically associated with increased ROS production. This, in accordance with other studies, shows that the anti-oxidative effects of Nrf2 are only seen at certain concentration levels^[147]^. Loss of function (LOF) of CCM1 results in increased JNK signaling, which has been shown to lead to increased Nrf2 activity^[148,149]^. JNK inhibitors resulted in decreased Nrf2 activation and restored ability of autophagy. Impaired autophagy is seen in CCM lesions and rescue of this phenotype suggest that it is mediated by JNK signal pathway. Similar to Nrf2, supraphysiological concentrations of Glo1 result in increased sensitivity to oxidative stressors^[150,151]^. These cells also have decreased levels of heat shock protein (HSP), Hsp70 and Hsp27, which have a protective role in the cell by increasing cellular capacity to handle stressors. The sequential effects by Glo1 compound to increase sensitivity to stressors and decrease tolerance, result in greater propensity towards intrinsic cell death in CCM1 deficient condition^[152]^.

### Appropriate valvulogenesis is predicated on CCM1-CCM2 complex

The fluid stress on the EC cells is paramount for appropriate differentiation of the heart. It is shown that abnormalities in blood flow through alteration of KLF2a/b activity affect heg1. Heg1 is localized to areas of myocardium with increase fluid forces in zebrafish^[153]^. It was found the increased Heg1 expression stabilized Ccm1 in these cells. Therefore, overexpression of Heg1 resulted in increased Ccm1 leading to decreased Klf2a activity. This decreased Klf2a activity desensitizes the cells to the fluid forces resulting in appropriate valvulogenesis. In the absence of Ccm1, the endocardial cushions do not develop into functional valves. Induced expression of Ccm1 to endocardial cushions in *Ccm1* deficient zebrafish resulted in development of valves. These cells were shown to increase Notch signaling and decrease Klf2a activity with the targeted expression of Ccm1. Similar valvular defects were also seen with the inhibition of Notch activity^[154]^. Therefore, CCM proteins are involved in appropriate valve development^[155]^.

### CCM2 and CCM3 function coordinately in gonadogenesis

*Ccm2* and *Ccm3* gene expressions are upregulated in adult mouse testis and ovaries, correlating with CCM2 and CCM3 protein expression, suggesting the involvement of Ccm2 and Ccm3 in the regulation of gonadogenesis. The expression pattern of CCM2 changes through embryogenesis. In the prenatal testis stage, CCM2 is mainly expressed in the interstitial cells of Leydig with little expression in gonocytes. Throughout gonad maturation, CCM2 begins to be expressed in spermatocytes, followed by the expression of CCM3. In ovaries, CCM2 is found in the oocyte nucleus at birth. Overtime this expression is decreased while the expression of CCM2 is increased in adult granulosa cells. The CCM2 in granulosa cells is expressed solely in the cytoplasm based on the spatiotemporally differential expression patterns of CCM2 and CCM3 in the testis and ovaries; it is plausible that CCM2 and CCM3 proteins may have different physiological roles during testicular and ovarian development^[156]^. Homozygous Ccm3 mutants in a *C. elegans* model rendered them sterile. These Ccm3 mutant worms had multinucleated germ cells that showed hypoproliferation, which may be caused by altered expression of Rab-11. Ccm3 promotes endocytic recycling by interacting with Rab-11. Defective endocytic recycling could result in decreased expression of Glp-1, a mediator of Notch signaling, and Rme-2, a mediator of protein endocytosis. *Ccm3* deficient germ cells have defective late-stages of cytokinesis leading to multinucleate giant cells. Polarity of *C. elegans* is modulated by non-muscle myosin^[157]^, while non-muscle myosin distribution is regulated by Ccm3. *Ccm3*-null embryos have aberrant expression of Par-6 and Par-2, both of which are polarity proteins. Therefore, it can be concluded that embryonic polarity is mediated by Ccm3^[158]^.

### CCM2 plays a role in the cardiac phenotype seen in CCM

As we described before, CCM2 binds to MEKK3 through the HH domain, leading to increased expression in KLF2, KLF4, ADAMTS4, and ADAMTS5^[115]^. These expression changes can be detected in the earliest stage of CCM lesions. The increased KLF4 and KLF2 were not only found in CCM lesions but also blood vessels in the cerebellum in *Ccm1* knockout mice. These increased expressions were also reported in both sporadic and familial forms of human CCMs. Also, their early presence suggests that KLF4/KLF2 may be involved in the formation of lesions. MEKK3 is a MAP3 kinase that controls KLF2/KLF4 activity which is especially critical in cardiogenesis. Ccm1 null mutant mouse model is embryonic lethal, but Map3k3 haploinsufficiency is able to rescue this lethality. To determine the temporality of the Rho activation *vs.* MEKK3 activation, CCM1 depleted EC cells treated with hydroxyfasudil (a Rho inhibitor) was unable to normalize the levels of KLF4/KLF2 while normalized KLF4/KLF2 levels in CCM1 null cells resulted in normal level of Rho activation, suggesting that the KLF4/KLF2 signaling is upstream of the Rho activation in CCM lesions^[90]^. Increased KLF4/KLF2 expression can lead to increased ADAMTS that functions to cleave proteoglycan matrix such as versican. Therefore, the up-regulation of ADAMTS can lead to disruption of intervening parenchyma around the blood vessel resulting in the formation of a cavernoma^[12,13]^.

### CCM3 is a regulator of cell apoptosis

CCM3 has been linked to both apoptosis and cell survival pathways. Initially, CCM3 was discovered as a protein for granulocyte apoptosis. One proposed mechanism is that CCM3 binds with VEGFR2 resulting in increased stabilization of the receptor. Decreased CCM3 results in increased degradation of VEGFR2 leading to decreased VEGF stimulation^[135]^. Furthermore, CCM3 is implicated in translocation of MST4 to the periphery of the cell where it activates ERM proteins. These ERM proteins are anti-oxidative and thereby prolong cell survival^[159]^.

### CCM3 plays a role in exocytosis

CCM3 is known to interact with STK24 and UNC13D, a known vesicle fusion regulator, in neutrophils. STK24 is an inhibitor of neutrophil vesicle exocytosis. STK24 deficient neutrophils release larger amounts of enzymes through exocytosis. STK24 localizes to neutrophil granules. There are two pools of granules in neutrophils: readily available and reserved. STK24 is associated with increased release of the reserved pool. UNC13D is a protein that binds to vesicles to promote their exocytosis. STK24 inhibits UNC13D^[160,161]^. CCM3 has a dual effect on neutrophil exocytosis. CCM3 binds to STK24 and stabilizes it to increase neutrophil exocytosis. However, CCM3 also increases the binding of UNC13D to liposomes through a calcium mediated mechanism, which is only seen in high intracellular concentrations. This inactivates excess UNC13D, resulting in decreased exocytosis. Simply stated, CCM3 is important for maintaining equilibrium of neutrophil exocytosis. Loss of CCM3 increases exocytosis of granules in neutrophils. This has been shown in renal ischemia-reperfusion injury model where reperfusion resulted in increased damage, suggesting that CCM3 deficiency results in an increased oxidative damage due to neutrophil exocytosis^[162]^.

CCM3 controls EC proliferation. Weibel-Palade bodies are granules in EC cells that contain angiopoietin-2 (ANGPT2)^[163,164]^. ANGPT2 binds to a tyrosine kinase receptor, TIE-2, and regulates the formation of EC cell-cell junction in angiogenesis. *Ccm3* knockout mice were shown to have increased Angpt2 expression. Furthermore, TIE-2 showed more phosphorylation in areas such as cerebellum and retina, areas classically known to form CCM lesions. As explained previously, CCM3 is a mediator of exocytosis in neutrophils through UNC13B. It was seen to mediate exocytosis in EC as well. EC cells were shown to have increased exocytosis of granules which can be rescued by the suppression of UNK13B. ANGPT2 transcription or translation was not affected by *CCM3* silencing, suggesting that this is not regulated at the transcriptional level. This is consistent with the theory that CCM3 blocks of exocytosis of ANGPT2. A decreased CCM lesion burden was observed in *Ccm3* knockout mice with the introduction of ANGPT2 antibodies, reaffirming the involvement of TIE2 signaling in the CCM lesion formation. This finding provides another venue for potential pharmacotherapy^[90]^.

### CCM3 might have multiple cellular functions through its partners

CCM3 interacts with STK25 or MST4 to form the STRIPAK complex^[165]^ which localizes to the cis-face of the Golgi complex^[61]^. At this location, it plays a role in appropriate positioning of the Golgi. GCKIII kinases are activated by homodimerization and resulting in its autophosphorylation, but activation is tightly regulated by CCM3^[95]^. Dysfunction of CCM3 results in the malposition of Golgi complex and centrosome^[166]^. Migration is essential for proper placement of EC cells during angiogenesis. Increased expression of CCM3 causes over migration of EC cell^[162]^. Therefore, dysfunction of this process could be involved in the formation of CCM lesions.

### CCM3 stabilizes intracellular bridges

Certain cells such as germ cells have cytoplasmic connections that regulate cell-cell communication and coordination. Anillin proteins such as ANI-1 and ANI-2 regulate the length of these projections. ANI-1 is known to decrease bridge length, while its antagonist, ANI-2, increases bridge length^[167]^. It was found that GCK-1 that is regulated by CCM3 binds to ANI-1. Therefore, deficiencies in CCM3 and GCK-1 result in a decrease in intracellular bridge size. This results in multiple histological defects in the gonads such as reduced distal arm length, rachis diameter, and brood size. Fluorescence imaging studies showed that CCM3 localizes to the bridges. Co-deletions of CCM3/GCK-1 and ANI-1 resulted in increased bridge number, suggesting a similar pathway between GCK-1/CCM3 and ANI-1. Non-muscle myosin II (NMMII) is responsible for constriction of bridges. However, unopposed activation of NMMII causes hyperconstriction and results in destabilization of bridges^[168]^. CCM3/GCK1 deletion resulted in increased localization of NMMII to the intracellular bridges and ANI-1 binds to NMMII. Therefore, it was postulated that intracellular bridges is regulated by CCM3-GCKI-ANI-1-NMMII signaling cascade. Yet, co-deletion of these genes did not affect bridge size. Therefore, it is likely that GCK1/CCM3 affects intracellular bridges through other signaling pathways.

### CCM lesions have defective autophagy

CCM3, along with CCM1 and CCM2, are involved in many signaling pathways that result in increased production of ROS: Sirt1/FoxO1, JNK/c-JUN, β-catenin, and TGF-β pathways^[146,169–171]^. This oxidative stress will damage organelles in the cell. However, inadequate autophagy mechanisms hinder cell recovery ability leading to progression of disease. CCM lesions show defect in autophagy through increased activity of mTOR. Inhibitors of mTOR were shown to reverse the defect in autophagy suggesting that mTOR is involved in the process, which provides another set of pharmacotherapeutic agents in CCM.

### CCM lesions have differentially expressed miRNA

The composition of micro RNAs (miRNAs) in CCM lesions was analyzed through an mRNA expression screen. These results were supported by RT-qPCR. Compared to normal controls, it was found that 10 miRNAs were upregulated and 42 miRNAs was downregulated in CCM lesions. A more stringent analysis showed 5 miRNAs that were very significantly downregulated. Using bioinformatics, potential binding mRNAs to these 5 miRNAs were identified. One of the miRNAs had a potential 981 binding partners. Several proteins already implicated in CCM lesions were found to be targets of these miRNAs including MLLT4, VEGFA, MAPK1, RAC1, RHOA, FOXO1, ENG, SMURF1, and HEYL^[17,83,99,106,126,139]^. It was concluded that three miRNAs (let-7b-5p, miR-361-5p, and miR-370-3p) can potentially be involved in the pathogenesis of CCM^[172]^.

### CCM3 was frequently implicated in tumorigenesis

CCM3 was initially identified as a tumor-associated apoptotic protein^[21]^. Several cases of meningiomas have been reported in patients with dysfunctional CCM3, suggesting that CCM3 could potentially act as a tumor suppressor^[31,173,174]^. One report stated that CCM3 deficient EC cells can continuously proliferate in cell cultures. In fibroblasts, CCM3 deficient cells can grow several more generations before entering senescence, comparing to wild-type cells. This suggests that the depletion of CCM3 delays cell senescence. Gene enrichment analysis showed a decreased production of cytokines in CCM3 deficient EC cells. Cytokine production was not inducible with TNF-α in these cells. It was found that these cells have a defect in C/EBPβ activity. C/EBPβ expression was upregulated in CCM3 deficient cells, which delays the progression of cells into senescence. Therefore, the lack of C/EBPβ activity is the likely driven factor in delaying the cells into senescence. Gene enrichment analysis showed decreased expression level of lysosome gene set. Senescent cells have increased autophagy for unutilized organelles and CCM3 deficient cells do not show increased activity of autophagy. Growth of CCM3 depleted cells in minimum nutrient media showed impairment of autophagy. Therefore, CCM3 deficiency increases C/EBPβ activity that, in turn, impairs cell senescence, resulting in declined cellular autophagy^[175]^. More research is needed to elucidate the underlined relationship between CCM3-mediated senescence and meningiomas.

CCM3 mRNA 3’UTR was found to be able to bind to Mir-103, a microRNA that was found to be associated with prostate cancer. This microRNA was found to be down-regulated in prostate cancer. Furthermore, *in vivo* studies showed that up-regulation of Mir-103 restored cells to senescence. The *in vitro* studies of cell culture showed that Mir-103 plays an important role for G1/S cellular checkpoint. When Mir-103 binds to CCM3 mRNA, it targets the CCM3 transcript for degradation. The decreased expression of CCM3 in the cell leads to increased apoptosis. Further, overexpression Mir-103 in normal cells resulted in increased apoptosis. Likewise, down-regulation of CCM3 observed in a prostate cancer cell line resulted in increased apoptosis^[176]^, suggesting that CCM3 may play a role as oncogene or tumor suppressor (depending on cell signaling), in the tumorigenesis.

**Figure 1:**
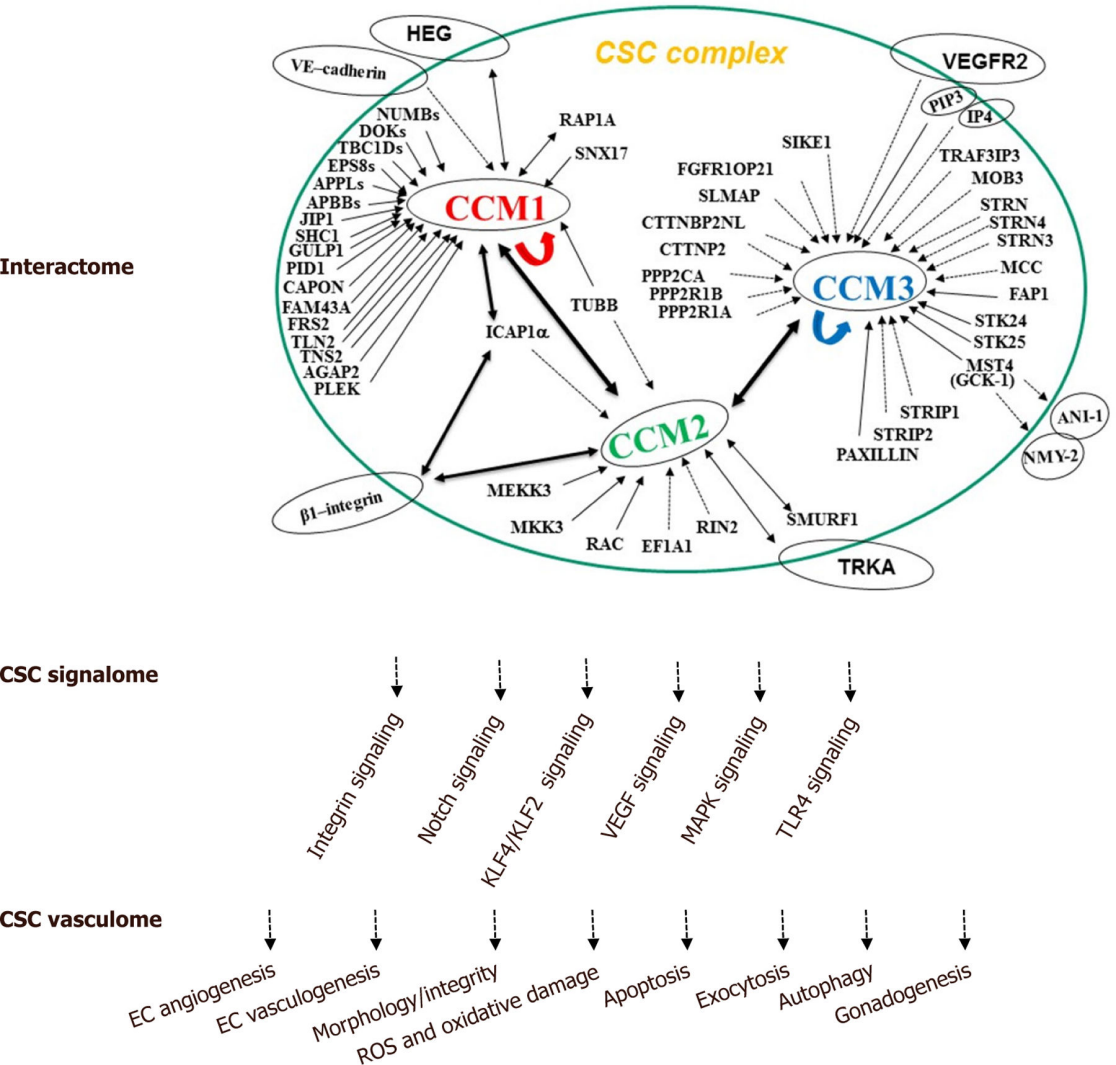
The cellular roles of the CSC complex. The schematic diagram summarizes the known CSC interaction protein partners (interactome), defined CSC-modulated signaling pathways (signalome), and distinct molecular and cellular functions of CSC complex (vasculome) with our current understanding of CSC cellular functions. CCM: cerebral cavernous malformations; CSC: CCM signaling complex; EC: endothelial cells

In summary, CCM proteins form a signaling complex (CSC) that have been demonstrated to play major roles in the regulation of multiple cell structures and signaling mechanisms involved in fundamental physiological and biogenic functions, as well as in cell responses to various environmental stressors. We have detailed these recent findings in the review, with a diagrammatic summary of major functions of CSC in three categories: interactome, signalome, and vasculome [Figure 1].
